# Sustaining our rural allied health workforce: experiences and impacts of the allied health rural generalist pathway

**DOI:** 10.1186/s12913-024-11207-5

**Published:** 2024-06-19

**Authors:** Alison Dymmott, Stacey George, Narelle Campbell, Chris Brebner

**Affiliations:** 1 College of Nursing and Health Sciences, Flinders University Caring Futures Institute, Flinders University, Adelaide, South Australia Australia; 2 College of Medicine and Public Health, Flinders University Northern Territory, Flinders University, Darwin, NT Australia

**Keywords:** Allied Health, Rural Generalist practice, Rural and remote, Qualitative, Workforce, Education and training

## Abstract

**Introduction:**

Rural and remote communities face significant disadvantages accessing health services and have a high risk of poor health outcomes. Workforce challenges in these areas are multifaceted, with allied health professionals requiring broad skills and knowledge to provide vital services to local communities. To develop the expertise for rural and remote practice, the allied health rural generalist pathway (AHRGP) was introduced to develop and recognise specialist skills and knowledge required for rural and remote practice, however the experiences of professionals has not been explored. This study gained the experiences and perceptions of allied health professionals undertaking the pathway as well as their clinical supervisors, line managers, profession leads and consumer representatives.

**Methods:**

A qualitative study was undertaken drawing on pragmatic approaches across four research phases. This study was one component of a larger mixed methods study investigating the experience, impact and outcomes of the AHRGP across six regional Local Health Networks in South Australia (SA). Interviews, surveys and focus groups were conducted to explore the perceptions and experiences of participants. Data was analysed thematically across participant groups and research phases.

**Results:**

A total of 54 participants including 15 trainees, 13 line managers, nine clinical supervisors, six profession leads, four program managers and seven consumer representatives informed this study. Five themes were generated from the data; gaining broad skills and knowledge for rural practice, finding the time to manage the pathway, implementing learning into practice, the AHRGP impacts the whole team and confident, consistent, skilled allied health professionals positively impact consumers.

**Conclusion:**

The AHRGP is offering allied health professionals the opportunity to develop skills and knowledge for rural and remote practice. It is also having positive impacts on individuals’ ability to manage complexity and solve problems. Findings indicated consumers and organisations benefited through the provision of more accessible, consistent, and high quality services provided by trainees. Trainees faced challenges finding the time to manage study and to implement learning into practice. Organisations would benefit from clearer support structures and resourcing to support the pathway into the future. Incentives and career advancement opportunities for graduates would strengthen the overall value of the AHRPG.

**Supplementary Information:**

The online version contains supplementary material available at 10.1186/s12913-024-11207-5.

## Introduction

People living in rural and remote areas face significant inequalities in access to health care in comparison to metropolitan areas [1, 2]. They also have a higher risk of experiencing disease and poorer health outcomes [3, 4]. In Australia specifically, rural and remote populations experience wide ranging and complex health conditions over vast geographical areas with limited access to health professionals to support them [1]. Previous studies have explored strategies to address workforce challenges in rural and remote areas, but these predominantly address doctors’ needs [5, 6]. Medical and allied health professionals face different challenges and opportunities early in their career in rural areas. Doctors are immersed in structured training programs with associated career advancement incentives. In contrast allied health professionals post graduate training is variable and not directly related to career advancement. Further, supervision methods, organisational support and personal circumstances are variable for both groups. Different workforce strategies are required for both groups accordingly [7].

Allied health professionals working in rural and remote areas provide vital and wide-ranging services to local communities [8]. Allied health is a broad professional term encompassing many individual professions who are not part of the medical, dental or nursing professions. They provide a broad range of diagnostic, therapeutic and technical services with the aim of improving function, health and wellbeing of consumers [9]. Until recently there were limited opportunities for allied health to be recognised for advanced skills in rural and remote practice. Further, career advancement opportunities and adequate retention strategies have limited exploration in the literature [10]. However, there is increasing recognition that structured training and professional support assists rural and remote allied health professionals to develop and maintain skills that advance their careers [7, 11, 12]. In the Australian context, the Allied Health Professions Office of Queensland paved the way for postgraduate training and workforce development initiatives by introducing allied health rural generalist training positions from 2013 to develop and recognise the specialist skills and knowledge required for rural and remote practice [13] and to improve recruitment and retention outcomes. Over the following four years, the Allied Health Rural Generalist Pathway (AHRGP) was established which included a formal post graduate training program, workforce structures, supports and the development of rural generalist service models and competencies [14], with other states and jurisdictions subsequently offering the AHRGP.

In 2017, James Cook University (JCU), in the state of Queensland Australia, introduced the rural generalist program (RGP) [15] which became a core component of the AHRGP. The RGP is a two-level post graduate program (https://www.jcu.edu.au/courses/graduate-diploma-of-rural-generalist-practice). Level 1 is a non-award program designed for early career rural or remote allied health professionals with less than 3 years of experience. It comprises 12 modules that are each 6 weeks in duration requiring approximately 22 h each to complete. The level 2 program is a graduate diploma qualification, with eight modules requiring approximately 130 h per module and is designed for rural and remote allied health professionals who have more than 2 years of work experience [15]. Both levels offer specialist training and education designed to support clinicians working in rural and remote areas and incorporate online coursework and service development work based projects and activities [15]. While participating in the AHRGP, trainees are afforded dedicated study time and clinical supervision at work and the opportunity to participate in service development or quality improvement project work.

Research investigating the experience and impact of the AHRGP has identified emerging positive outcomes for trainees undertaking the pathway, employing organisations and communities [13, 14, 16–19]. In the first evaluations when the AHRGP was under development, Queensland Health found that allied health professionals in rural generalist training positions’ job satisfaction improved, and they gained relevant skills for practice through their participation [18]. Health services with training positions reported their capacity to manage clinical workloads and volume of quality improvement activities undertaken increased, along with multidisciplinary team communication and coordination improvements. It was also reported that consumers had decreased need to travel, quicker access to health services and improved quality and continuity of care [13, 18]. The first cohort of trainees had a 100% completion rate and 78% remained working in rural or remote locations six months after completion of the AHRGP [13, 14]. This early research was undertaken prior to the introduction of the RGP and the training positions were supernumerary. A study in another state of Australia, New South Wales, was undertaken after the RGP was established, however the number of participants was small and were all from one discipline with just one completing the RGP. The findings of that study were that the pathway enabled services to recruit and retain physiotherapists into positions that had previously been difficult to fill. This had a positive impact on both service and consumer outcomes [17].

Two recent studies have examined the RGP specifically [16, 19]. Through including participants who were enrolled in the training program with JCU, the researchers were able to examine the program impacts across multiple jurisdictions. The study identified a range of benefits and challenges of the program for trainees and employing organisations [16], concluding that the program is assisting in building capacity of allied health professionals working in generalist roles and more broadly, a rural allied health workforce [19]. Benefits were also identified for consumers including increasing accessibility and quality of services received [16, 19]. The findings of these studies were important in identifying the specific outcomes of participating in the RGP as this was not the focus of the previous studies.

In 2019, Rural Health Workforce Strategy funding provided by the Government of South Australia supported a trial of the AHRGP in SA Health regional local health networks (LHNs). Significant gaps in the research existed in relation to the AHRGP. These included investigating the impact of the whole pathway including the RGP but also the supports and opportunities provided by the workplace, a synthesis of experience of rural generalist trainees, employing organisations and consumers receiving services, the workforce outcomes and costs of implementing the AHRGP and the contextual factors for successful implementation of the AHRGP.

Addressing these gaps in knowledge provided evidence to inform future implementation of the AHRGP to maximise retention of AH staff in rural health services. The aims of this research were to comprehensively examine the experience, impact and perspectives of the AHRGP for trainees, organisations and consumers as it was introduced in rural South Australia. Specifically, the aims were:

1. To explore the experience of the allied health professionals participating in the AHRGP and the impact on their skills, abilities and knowledge for practice.
2. To understand the impact and experiences of the AHRGP from the perspective of clinical supervisors, profession leads and line managers working with rural generalist trainees.
3. To explore how the AHRGP has impacted employing organisations and consumers receiving allied health services.

## Methods

This paper reports on the qualitative data collected as part of a larger multi-level, multi-phase pragmatic mixed methods research project. This study took a constructivist approach where the researchers consider multiple realities constructed by individuals and influence by social factors [20]. With this approach, the researchers were able to consider the views, perspectives and experiences of a wide variety of participants to explore the complexity of rural workforce and training to look at the research questions from multiple perspectives to generate rich, meaningful findings and connections. Ethics was gained by the Southern Adelaide Clinical Human Research Ethics Committee 21 August, 2019 HREC/19/SAC/170. All methods were performed in accordance with the relevant ethical guidelines and regulations.

### Sampling

This research took a purposeful sampling approach to select participants. SA Health provided the researchers with the contact details of all trainees who were participating in the AHRGP as well as their individual clinical supervisors, line managers, profession leads and program management team (herein referred to collectively as service leaders) and consumer representatives who were members of the LHN Advisory Board, in which the trainees were working. Clinical supervisors and line managers were allocated to trainees by the organisation according to work role and location. Profession leads and project managers provided leadership, coordination and support across all regional LHNs. A clinical supervision framework guided the provision of supervision [21] and individual LHN determined management structures and roles. All trainees involved in the pathway were invited to participate in the research via an email invitation as well as their associated service leaders, the consumer representatives and the program management team overseeing the pathway introduction. Thus, there were three participant groups. Informed consent was gained prior to data collection.

### Data collection methods

This research was undertaken over four research phases, the methods used are outlined in Table 1. Semi structured interviews were conducted face to face or via teleconference by the chief investigator and were scheduled with each trainee as they reached the associated milestone in the pathway (i.e. beginning, the midpoint and endpoint). The semi-structured nature of the interviews enabled the researchers capture individuals’ experiences and to build on the generated themes in subsequent interviews.

Service leaders’ interviews were scheduled around the same time as their associated trainee. Interview and focus group questions were developed using Kirkpatrick’s four levels of training evaluation [22], consultation with the project management team, and the review of other relevant literature, and can be found in the supplementary material. Focus groups were conducted via teleconference with the chief investigator and an additional researcher (CB) and were undertaken in 2019 when trainees were starting the pathway, and in 2022 when the trainees had completed the pathway. In the first focus group, consumer representatives were asked to describe what a quality allied health service would look like for their communities. In the second focus group the consumer representatives were presented with the preliminary research findings from this study and asked to discuss how they envisaged the findings would impact consumers in their LHNs. Teleconferencing was utilised due to the vast distances in which focus group members were located across. Consumer representatives were supported by LHN staff to access the facilities. A follow up survey was also sent to trainees 6 months after completion. All interviews and focused groups were recorded, deidentified and transcribed for analysis. Survey data was combined with interview transcripts for coding.

**Table 1:** Research phases and associate methods

Research phases	Qualitative data collection methods for participant groups
**Phase 1** – Pre pathway 2019	Trainee interview Clinical supervisor, line manager, profession lead and program manager interviews (service leaders) Consumer representative focus group
**Phase 2** – Mid pathway 2020	Trainee interview Service leader interviews
**Phase 3** – End pathway 2020-22	Trainee interview Service leader interviews Consumer representative focus group
**Phase 4–6** month post pathway completion 2021-23	Trainee follow up survey

### Data analysis

Thematic analysis of data was conducted across the 4 research phases [22]. Interviews and focus groups were transcribed by a third party and checked by the chief investigator for accuracy. The transcripts were read and coded inductively by participant group (trainees, supervisors, managers, advanced clinical leads and project managers) and by research phase using Nvivo (Lumivero; https://lumivero.com). Codes were named as close to the actual quote as possible to ensure they were attributed to the actual statements and not misinterpreted later during analysis. The first two interview transcripts were coded by a second researcher (CB) and codes were compared for consistency. A high level of consistency in coding and naming was found and subsequently the remaining transcripts were coded by the chief investigator and reviewed by the team. Once the coding was completed, the findings were analysed thematically by phase [22].

Firstly, the codes were synthesised for meaning by participant group to form categories by the first author. These categories were then collated across participant groups. Codes within categories were diverse in content due to the wide range of participant perspectives involved. The categories were thoroughly discussed with all authors at fortnightly meetings between 2020 and 2022 at each phase as new data was collected and coded. The research team identified similarities and differences in categories across participant groups and phases [23]. Qualitative findings from the phase 4 surveys were also incorporated into categories at the conclusion of data collection in 2023. As the survey was brief, the responses were not coded extensively but were checked against established codes and categories for any new or different perceptions 6 months post completion. At the end of the final phase, the categories and findings from all four phases were brought together to identify patterns. All authors worked together to generate themes at each research phases to ensure rigor. As each new phase was completed the themes were confirmed or modified as new data was collected. Using thematic analysis gave the analysis structure and ensured transparency [24]. Codes and categories that fell into multiple themes were thoroughly discussed by the research team to explore their meaning to determine their best fit. Some overlap in themes categories and codes were difficult to extrapolate considering the large volume of data, for example the notion of time fell into two themes.

## Results

This research was conducted between September 2019 and June 2023. A total of 54 participants were involved in the study including: 15 trainees across five allied health professions and six rural regions in South Australia. In addition, 13 line managers, nine clinical supervisors, six profession leads, four program managers and seven consumer representatives participated in this study. Not all service leaders and consumer representatives were involved in all phases due to availability and staffing changes. Nine of the trainees participated in the level 1 RGP and five were in level 2. See Table 2 for details of participant involvement. Of the 15 trainees who participated, seven completed the AHRGP and one was continuing beyond the end of phase 4. Three of the nine level 1 trainees completed the pathway and four of the five level 2 trainees had completed the pathway on the completion of this study with one continuing beyond the end of the research period. INSERT Table 2 HERE.

**Table 2:** Research participant demographics

	Total number of participants	Phase 1	Phase 2	Phase 3	Phase 4
Level 1 trainees	10	9	8	4*	3
Level 2 trainees	5	4	5	5*	4
Occupational therapy trainees	4	4	3	3	3
Physiotherapy trainees	3	3	3	2	2
Podiatry trainees	4	4	3	3	1
Speech pathology trainees	3	3	2	1	1
Social work trainees	1	1			
Clinical supervisors	9	9	9	7	
Line Managers	13	7	6	9	
Clinical leads	7	4	4	5	
Consumer representatives	7	5		4	
Program managers	4	3	3	4	

* Two of the trainees in phase 3 had not completed the pathway but participated in phase 3 as they left between midway and the end

### Thematic analysis of findings

Five themes were generated from the data across the four research phases and 54 participants.

1. Gaining broad skills and knowledge for rural practice.
2. Finding the time to manage the pathway.
3. Implementing learning into practice.
4. The AHRGP impacts the whole organisation.
5. Confident, consistent, skilled allied health professionals positively impact consumers.

### Theme 1: Gaining broad skills and knowledge for rural practice

Throughout the four research phases, participants described trainees gaining knowledge, skills and confidence to work as rural generalist allied health professionals. These gains had similarities and differences across the four phases. In phase 1 as the trainees were beginning the AHRGP they anticipated the learning and skills development they would gain including the development of confidence and skills to work as a rural generalists. They were also hoping to improve service delivery for their organisations and consumers. In phase 2, halfway through the pathway, trainees reported gaining broad and specific skills and knowledge for practice, specifically, they discussed the benefits of learning program management skills, understanding how the organisation operated and the scope of rural generalist practice.

*“I guess the big thing would be the quality improvement stuff. That’s probably changed my practice just in terms of I think sometimes as a new graduate you’re keen to contribute to things, and you kind of maybe see, like, a gap at your site that you can contribute to. And I definitely probably now understand the process that goes behind that and who to kind of talk to.” Trainee 8 phase 2*.

In phases 3 and 4 when the trainees had completed the AHRGP, reports of the attainment of confidence in their work roles, taking risks and advancing their career were identified. Trainees also reported broad skills they had gained including evidence-based practice, knowledge for generalist practice, leadership skills and operational knowledge.

*“I think there’s skills I’ve gained in this that I wouldn’t have otherwise ever gained. And the development and career opportunities that it’s really opened up. Like I couldn’t do the job that I’m in at the moment if I didn’t have (AHRGP), and I probably wouldn’t have gotten, or been prepared for my last role either.” Trainee 12 phase 3*.

In phases 2 and 3, service leaders described the ways in which trainees were developing their confidence and skills in providing allied health services across a broad range of service types with more autonomy. In particular, in phase 2, service leaders reported trainees were able make clinical decisions more easily and manage high levels of complexity which they saw as hugely beneficial.

*“I think they’ve shown a high level of understanding in some really tricky situations, and I think they just … I don’t think they flinch much around that. They are happy to lay out their understanding and their reasoning and what they would recommend and they’re happy to take on feedback. But all in all, they do a really good job of making those decisions.” Service leader 23 phase 2*.

Additionally, in phase 3, service leaders described trainees developing system and strategic thinking in the later stages of the AHRGP. They also noted trainees were developing their leadership skills and were advancing their careers.

*“I think on a broader scale they have been able to apply the learnings to their overall role and career progression really within their team and leadership progression” Service leader 20 phase 3*.

### Theme 2: Finding the time to manage the pathway

Time was a theme that was generated from the data in every phase and with all stakeholders. Although the AHRGP was beneficial, trainees found it challenging to find the time to manage the study commitments, to find a work life balance and to maintain motivation over an extended period.

While undertaking the AHRGP, trainees were assigned half to one day a week to undertake study related activities at work. In each of the four research phases, trainees reported the challenge of setting this time aside. The challenges related to staff vacancies in teams, heavy workloads, interruptions, unpredictable clinical work, administrative follow up work and the attainment of leadership roles with higher levels of responsibility. Although trainees were given permission to undertake study time at work, this time was not backfilled, and trainees and service leaders reported it was difficult to prioritise study time over clinical priorities. Trainees also travelled extensively for work which impacted their ability to find time to study.

*“… A lot of the clinicians really struggled to, sort of, balance that with, “Oh, we’ve got 25 more people on the waiting list but I’m supposed to study.” And that balance between doing what they thought they should do, and that work ethic, versus the ethic of participating in the programme” Service leader 24 phase 2*.

Work life balance was described as challenging for trainees participating in the AHRGP. Relating to the difficulty of finding time to study at work, there was a need to undertake assignments and coursework in trainees’ own time.

*“I tried everything in the book to try and have a separation between work, study, life, but you get home from work, I’m exhausted, I can’t do study, that leaves the weekend. And when you’ve only got two days in a weekend and I get one day of work to do it, to do two subjects in that time, it barely fits.” Trainee 13 phase 3*.

The challenge of maintaining motivation throughout the AHRGP was raised by trainees in the second half of the pathway (phases 2 and 3). Some trainees reported it was challenging to maintain motivation and stay focused to study over an extended period of time, while others found the pathway took more of their time than they had anticipated. Service leaders also noticed trainees struggled more with the in the second half of the pathway. It is important to note that some trainees completed the pathway more quickly than others and that the level 2 pathway generally took longer to complete than level 1.

*“At the start it was all new and interesting and I really enjoyed it as a break from the clinical side of things for the first half of the program, whereas found the second half a lot more challenging to just keep focused and to prioritise it and see it helping me and relating to my practice as a motivator.” Trainee 4 phase 3*.

### Theme 3: Implementing learning into practice

Participants described a range of experiences in terms of implementing learning into practice during the AHRGP. Some trainees were able to easily apply the learning materials to their practice and work roles, while others found it more challenging. Being able to implement learning into practice was related to the relevance of RGP course material to trainees’ work context, the opportunities to implement service development project work and motivation and incentives to complete the pathway.

Relevance of course material was related to topics undertaken, the professional background of the trainee and the type of work they were undertaking. The RGP has a combination of mandatory and elective topics. Some trainees and supervisors described the wide range of topics available as being informative and relevant to their rural allied health context and while others found the program contained less relevant clinical presentations and assessment tasks for their work context. Participants recognised that it would be challenging for an education provider to cater to the needs of multiple professions that was relevant to everyone and they described a range of ways in which JCU had provided a flexible approach to trainee’s learning. In particular, speech pathology and podiatry participants and trainees working in specific areas of practice rather than across a broad scope of practice found the material challenging to apply at times.

*“You look at the resources, there’s two for podiatry and one for pharmacy, and then there’s eight for physio … it’s very obviously weighted that way, I think.” Trainee 11 phase 3*.

Occupational therapists and physiotherapists were generally able to apply the course materials to their work and identify opportunities to implement their learning with consumers they were working with.

*“I think it enabled me to learn about a lot of different topics that I probably wasn’t really aware of before…. I think it helped me to integrate into my role and to learn a bit more about where I fitted and what OT can provide. Yeah, just broaden my horizons a little bit more.” Trainee 10 phase 3*.

The attainment of project management and evidence-based practice skills was found to be beneficial broadly across participant groups. Trainees felt the AHGRP enabled them to develop these skills more quickly and comprehensively than they would have without the pathway.

*“I’ve kind of got those skills now where I can identify an issue in the workplace and be like, alright, I’m going to do a bit of evidence-based research on this and then I’m going to see how I can kind of plan to improve that service” Trainee 13 phase 2*.

The challenge of implementing and evaluating planned projects and other work integrated assessments tasks was also widely reported across phases. Building on the findings in theme 2, trainees felt the limited time they had assigned to study at work prevented them from implementing their learning and service development projects into practice. For example, one trainee developed a plan for implementing a new group program for consumers but was unable to implement in her service and another trainee developed a project plan for a telehealth service but did not implement it. Further, some participants found the expectation to produce service development project outcomes while undertaking the coursework was overwhelming. Some service leaders described the lack of implementation of service development projects as reducing the overall impact of the AHRGP.

*“I really struggle with this idea that somehow, like you’re in it to learn, and then somehow at the same stage, and I get you’ve got to have outcomes and measurables and stuff like that, like you’re just learning at the same time” Trainee 12 phase 3*.

Motivation and incentives for completion of the AHRGP were barriers identified by some participants impacting their ability to implement learning and benefit from the pathway. Some trainees’ reported it was difficult to feel motivated to do coursework that didn’t feel directly related to their clinical work or that they needed to modify to implement into practice for example a project plan that did not meet the requirements of an LHN’s policy but that was required for an assignment. o. Implementing learning into practice and maintaining motivation were further inhibited by the lack of correlation of the pathway with career progression incentives and recognition of advanced skill or scope of practice.

*“I’m not say that coming out of this I feel like I should be all of a sudden titled differently or something like that but you don’t have the wolf behind chasing you to get to the finish line because it’s a nice thing to have accomplished and learnt things along the way but it doesn’t actually change, well it doesn’t feel like it changes anything significantly, move you up to a different position or something like that.” 4*.

### Theme 4: The AHRGP impacts the whole organisation

Service leaders identified a range of positive outcomes for themselves, their teams, and the whole organisation. There were also at times, challenges experienced in providing support to trainees.

During phases 2 and 3, service leaders identified a range of ways in which the organisation was impacted by the AHRGP. Trainees were being retained for longer in regions which had positive flow on effects for the whole team. Service leaders felt the pathway was giving trainees a reason to stay in the region, helping them develop their career and demonstrating that the regions had a culture of learning.

*“Yeah, I think we get better outcomes for our communities if we’ve got confident, competent staff that are here for the long run, and overall, that helps to build a stronger team, because your team morale and everything increases, if you’ve got happy and confident skilled staff to work with.” Service leader 28 phase 2*.

The overall skill level of teams improved through trainees gaining skills, being able to manage a broader clinical load which was imperative for rural practice. Service leaders also reported trainees were sharing their knowledge and skills with colleagues within and across regions which resulted in broader organisational benefits.

*“We’ve got very junior staff, so how they are able to come in and to being able to guide them, mentor them, and assist them from that generalist point of view is really crucial, because it’s quite difficult when you’re a new AHP, and (name) been able to embed them in really smoothly.” Service leader 19 phase 2*.

Service leaders also recognised that the trainees were demonstrating leadership skills which resulted in them identifying service gaps and implementing quality improvement activities to improve service delivery. They were also moving into supervision and managerial roles as they progressed through the pathway.

*“So, it was really, from what I’ve seen, is how they’ve grown not just as a clinician, but as a leader … And how they incorporate the, I guess, the rural generalist skills knowledge into the leadership space, as well as just their own individual practice …they’ve got a really good position to actually really make some definite changes and implement the change … And make it sustainable, as well… ” Service leader 24 phase 2*.

The AHRGP requires services to provide dedicated clinical support to trainees during the pathway and this study found that both clinical and managerial support were valued by trainees. They described a range of examples where support from a supervisor or line manager had made a difference to their experience in the pathway. Trainees’ felt like they had someone to discuss the pathway with, to get advice and to work through assessment tasks and service development projects with. Service leaders also felt they benefited from working with trainees as they gained skills themselves and took pride in seeing the trainees grow and develop.

*“Yeah. Just she was a really good resource to be able to find things that I might have not known where to look. But she had a really good insight into that, so yeah, she knew that she’d seen things before so she sort of just followed where logically she would have put it, and found it and sent it all to me, which was good.” Trainee 10 phase 2*.

In some circumstances, it appeared that organisations were not able to provide the level of support that trainees required. Some trainees reported feeling inadequately supported by a supervisor or manager. Some service leaders also described challenges knowing how to support a trainee. Further, service leaders were at times, unsure how their LHN was benefiting from the AHRGP. These challenges generally arose for service leaders who had not been in their role when the trainee was selected for the AHRGP and missed the initial phase of the AHRGP introduction or had previously not worked directly with early career allied health professionals.

*“I didn’t have any, any expectations put on me of what I needed to be doing with them. So, I, you know, I haven’t checked in with them and now I’m feeling really bad. But yeah so I think maybe some more organisational stuff around that.” 47 phase 3*.

### Theme 5: Confident, consistent, skilled allied health professionals positively impact consumers

Consumer representatives, service leaders and trainees identified a range of ways in which consumers were impacted by the AHRGP. The improving skills, confidence and knowledge of AHRGP trainees had positive impacts on consumers. With trainees being retained in the LHNs longer, participants felt consumers would have access to better quality care from consistent allied health professions who knew them and could provide more client centred services.

In phase 3, trainees felt they had gained skills to be able to better meet the needs of consumers, they felt more able to manage complexity and solve problems for consumers. They reported having the opportunity to learn more about specific clinical conditions that were relevant to consumers they were working with to identify new and effective assessment and intervention modalities. Trainees also described the services and projects they had developed that they felt would have indirect benefits for consumers in their regions.

*“[The AHRGP] Helped increase the sort of efficiency and the consistency, and the longevity of some of our rehab programs. So, that will help some of our community members now and in the future.” Trainee 3 phase 3*.

Service leaders identified a range of ways in which consumers were impacted by the AHRGP in phases 2 and 3. They felt that trainees were using more evidence based practice with consumers and that they were more confident and skilled in their service provision. Trainees were able to provide a wider range of services to meet consumers’ needs and the quality of care was improving.

*“If you think about an overall service, I think our quality has gone up, we’re actually meeting the need of the consumer much more effectively than maybe we were before with these staff because they’re able to do whatever we need them to … I think because they’re both advanced in their skills … the quality of service that they’re now being able to supply is increased.” Service leader 18 phase 3*.

Consumer representatives described rural generalist skills as being imperative for rural practice in their LHNs and they felt having more trained generalists in the region would result in less travel to services for consumers. They also felt more trained staff were more likely to be retained in the region which would have positive impacts on the consistency of service delivery.

*“… If you’ve got the higher trained staff in the area, but people don’t have to go then to the city, and we can … and that’s what we’re trying to do in our region. Keep as many people in our region as we can because we can provide the services.” Consumer rep 55 phase 3*

*“Not just looking at it from this isolated point of view but looking at it holistically. I guess that’s the catch word, isn’t it? We’ll look at it holistically and make it a whole package to keep families and keep people in areas which I would dearly love to see. Especially as I get older I want more people here that look after me”. Consumer rep 53 phase 3*

Consumer representatives felt the AHRGP could potentially benefit communities more if trainees received incentives to participate in the pathway which might improve the retention of trainees further. They also felt it was imperative that feedback from trainees in relation to the relevance of content should be taken on board to ensure the pathway can be as beneficial as possible.

*“And I think structurally, you really need to think about incentivising the whole program … I think there needs to be recognition of that in terms of remuneration or the level that those people come in at. I think that if you’re going to do that, we need to make sure that there is an incentive for people to do that. Either that they get promoted to the next level or there’s some kind of other financial incentive.” Consumer rep 54 phase 3*.

## Discussion

This large study described a wide range of perspectives and experiences of the AHRGP including allied health professionals who were participating in the pathway, clinical supervisors, line managers, profession leads and program managers who were supporting trainees and consumer representatives who experience allied health services.

The results demonstrate that the AHRGP had a positive impact on allied health professionals’ developing rural generalist skills and knowledge for practice. In particular, the development of evidence based practice and project management skills had positive impacts on practice. The AHRGP facilitated the development of evidence based practice through investigating clinical areas of interest and enabling trainees to use real life examples from their practice to deep dive into the best evidence for allied health assessment and intervention. They attained project management skills by developing quality improvement and service development projects for their workplaces while they learnt. These work integrated approaches are consistent with recent research demonstrating that allied health professionals are more likely to implement their learning from training into practice if they have the opportunity to practice their skills while they learn [25].

Through gaining broad skills and knowledge, trainees felt they were able to manage a higher level of clinical complexity. Service leaders found trainees had developed critical and flexible thinking skills to be able to manage complexity and solve problems. They also felt trainees developed a broader understanding of rural and remote practice through participating in the AHRGP. These skills are imperative for diverse workloads in geographically remote locations. Barker et al. also found trainees undertaking the RGP across Australian jurisdictions knowledge and skill improved in evidence-based practice, managing complexity and problem-solving [16].

Organisations employing allied health professionals can benefit from the AHRGP in a range of ways. Previous studies have identified a range of organisational benefits including improved: efficiencies, effectiveness and accessibility of services [16, 19]; workforce outcomes; and support mechanisms for allied health professionals [17]. The results of this research were consistent with previous findings as well as indicating that organisations benefitted from trainees sharing their skills with peers, which had a broader impact on the whole team. Conflicting findings emerged in terms of career advancement. Service leaders felt trainees were advancing their careers through the development of skills and knowledge in the pathway, they were able to take on more responsibility and support other staff. . In contrast, some trainees reported the absence of incentives or recognition of learning impacted their motivation to complete and was disappointing considering the effort they had put in. Overall, the AHRGP helped to build an effective rural and remote allied health workforce but further work is needed to incentivise completion.

Consumers benefitted from working with more skilled, knowledgeable, confident, and consistent allied health professionals. The AHRGP assisted trainees to develop skills and knowledge relevant to their clinical practice. Previous studies have considered the impact of the AHRGP on consumer experiences [19] and outcomes [17], but this study was the first to involve consumer representatives. This study found that consumer representatives thought the AHRPG was generating relevant outcomes for consumers and that organisations should continue to invest in the pathway.

Challenges for trainees identified in this research are comparable with those found in previous studies examining allied workforce strategies. These include protecting study time at work, workload pressures [17], higher than expected study load, irrelevant learning activities and time taken away from clinical work to study [16]. This research also identified additional challenges that had not arisen in previous studies. Some trainees experienced difficulties accessing adequate clinical and managerial support whilst undertaking the pathway. This had a negative impact on their experience of the pathway. The impact the AHRGP had on work-life balance and overall wellbeing was also widely reported in this study. Achieving a balance was especially challenging in the second half of the pathway when trainees were facing difficulties maintaining motivation to study. This new knowledge will be particularly helpful for organisations and allied health professionals considering the AHRGP as they establish study and support structures and routines to facilitate success.

To date, the AHRGP is offered in Australia and the authors did not identify any equivalent training program offered in other countries. Rural generalist medical pathways are offered internationally [26] with a range of favourable outcomes for rural based doctors, organisations and communities [27]. These pathways have allowed rural doctors to specialise in rural and remote practice with associated specialisation and career advancement opportunities [26]. Unfortunately, the current rural generalist pathway for allied health professionals is not directly associated with career advancement opportunities, renumeration incentives or the recognition of specialist skills. Trainees and consumer representatives in this study recommended the AHRGP have tangible completion incentives and appropriate remuneration for graduates to increase the value of the pathway.

This study identified challenges relating to implementing service development projects beyond the planning phase required for the RGP. Participants described a range of innovative projects they had designed to improve consumer and service outcomes and a desire to implement and evaluate them. Early research investigating the AHRGP described wide ranging benefits of trainees implementing service development projects, however the completion of the RGP was not as strong a focus [17, 18]. Organisations should consider addressing the potential barriers to project implementation which could improve stakeholder satisfaction and the overall outcomes of the AHRGP.

## Conclusion

This research has comprehensively examined the experience and outcomes of the AHRGP across multiple levels of stakeholders and over four distinct phases, which brings new knowledge and understanding for allied health professionals and employing organisations. Through exploring the experience of allied health professionals participating in the AHRGP, this study has identified positive positive impacts on trainees’ knowledge and skill for rural and remote practice. Challenges relating to the implementation of skills into practice and maintaining motivation require consideration for sustainability of the pathway. The perspectives of clinical supervisors, profession leads and line managers were extensively explored. They found the pathway enabled allied health professionals to gain skills and knowledge relevant for practice including solving problems, working across clinical areas and applying evidence based practice to their work. Although they found the AHRPG to be worthwhile, supervisors and managers would benefit from support and training in how to effectively support trainees in the pathway. This study explored pathway impacts for employing organisations and consumers. Consumer representatives identified a range of benefits including more consistent, high quality services for the community. Impacts for the whole service were significant including the developing of wide range skills across the whole service, retention of staff and development of leaders within the organisation. The value of the AHRGP could be improved through the development of associated workforce structures that recognise and reward the successful completion of the pathway and identify opportunities to implement service development projects and learning into practice more effectively. Managers and supervisors would also benefit from ongoing support to better understand how they can help trainees get the most out of the pathway. Considerations for how the course material can be more relevant for different professions and contexts would also be beneficial for success. Future research is required to explore the implementation of allied health rural generalist pathways in different contexts and internationally.

### Electronic supplementary material

Below is the link to the electronic supplementary material.

Supplementary Material 1

Supplementary Material 2

## Data Availability

The data that support this study were obtained from SA Health by permission cannot be publicly shared due to ethical and privacy reasons. Data may be shared upon reasonable request to the corresponding author if appropriate with permission from SA Health.
