# Supplementary material for: Sustaining our rural allied health workforce: experiences and impacts of the allied health rural generalist pathway
Source: BMC Health Serv Res. 2024 Jun 19;24:749. doi: 10.1186/s12913-024-11207-5 (PMC11186235; doi:10.1186/s12913-024-11207-5)
Supplement: Supplementary file 2 — Supplementary Material 2 [file 12913_2024_11207_MOESM2_ESM.docx]

## Allied Health Rural Generalist Pathway (AHRGP) Trainee pre pathway survey questions

Name

Age range 20-30, 30-40, 40-50, 50 and over

Profession

Local Health Network

Location

Position title

Classification

What type of contract are you currently on (permanent / temp (&end date); employment fraction (1.0/0.5Full time equivalent)?

Were you nominated by your region/ACL for the AHRGP, or did you initiate your application?

Specialty area or team if relevant

Did you grow up in a rural or metropolitan location?

How long have you been working as an AHP in a rural or remote location?

How long do you intend to work in a rural or remote location?

Why did you choose to participate in the AHRGP?

What goals are you hoping to achieve as part of the AHRGP?

What, if any, are your concerns with participating in the AHRGP?

Have you completed the Allied and Scientific Health Transition to Professional Practice Program? Yes, no, currently participating

Did your involvement in the Transition to Professional Practice Program in any way influence your decision to undertake the AHRGP?

Yes Definitely …………………………………………………………………………………………………..Definitely Not

How satisfied are you overall in your current job role?

| *Not at all satisfied* | | | | | | *Extremely satisfied* | | | | | |
| --- | --- | --- | --- | --- | --- | --- | --- | --- | --- | --- | --- |
| 0 | 1 | 2 | 3 | 4 | 5 | | 6 | 7 | 8 | 9 | 10 |
|  |  |  |  |  |  | |  |  |  |  |  |
| Currently in your role, how would you rate your CONFIDENCE as a rural generalist professional when: | | | | | | | | | | | |
| 1. Seeing patients/clients across the age spectrum (e.g. infants, children and adolescents, adults and older people)? | | | | | | | | | | | |
| *Not confident at all* | | | | | | *Extremely confident* | | | | | |
| 0 | 1 | 2 | 3 | 4 | 5 | | 6 | 7 | 8 | 9 | 10 |
|  |  |  |  |  |  | |  |  |  |  |  |
| 1. Delivering a large variety of services (e.g. health promotion, early intervention, acute hospital based services, sub-acute and ambulatory care services, chronic disease management, aged care, palliative care)? | | | | | | | | | | | |
| *Not confident at all* | | | | | | *Extremely confident* | | | | | |
| 0 | 1 | 2 | 3 | 4 | 5 | | 6 | 7 | 8 | 9 | 10 |
|  |  |  |  |  |  | |  |  |  |  |  |
| 1. Working across a variety of health settings (e.g. hospitals, health centres and clinics, patient homes, community venues) | | | | | | | | | | | |
| *Not confident at all* | | | | | | *Extremely confident* | | | | | |
| 0 | 1 | 2 | 3 | 4 | 5 | | 6 | 7 | 8 | 9 | 10 |
|  |  |  |  |  |  | |  |  |  |  |  |
| How would you rate your OVERALL CONFIDENCE as a generalist professional? | | | | | | | | | | | |
| *Not confident at all* | | | | | | *Extremely confident* | | | | | |
| 0 | 1 | 2 | 3 | 4 | 5 | | 6 | 7 | 8 | 9 | 10 |
|  |  |  |  |  |  | |  |  |  |  |  |

## AHRGP Trainee pre pathway interview questions

Why did you choose to work in a rural/remote location?

What are the best aspects of working in this location?

What are the challenging aspects of working in this location?

What factors are impacting on your intention to stay working in this location?

Are there any factors that, if enabled, would encourage you to stay longer?

The factors you have discussed can be classified as personal, organisational and professional, you mentioned……… considering this classification would you add any other factors that are important to you?

Thinking about the personal, organisational and professional factors you have mentioned how would you rate their importance in your overall satisfaction and intention to stay in this location?

Where are you hoping the pathway might lead or support you to do?

What will be some of the barriers and enablers of achieving your goals for the pathway?

What supports do you expect you will receive to achieve these goals and to assist you with the AHRGP?

What other supports do you think you will need as you begin the AHRGP? How do you envisage this could be provided?

What strategies are in place to ensure a minimum of half to one day a week in your workload is allocated to participation in the AHRGP?

## AHRGP Trainee midway survey questions

How far have you progressed through the AHRGP? How many modules have you done and how many do you have left?

How satisfied are you in relation to the overall AHRGP?

Not at all satisfied, not satisfied, neutral, satisfied, very satisfied

Are you consistently able to allocate a minimum of 0.1-0.2FTE to the AHRGP?

| Consider your average working week: approximately how much time per week was dedicated to your: |
| --- |
| Supervision |
| Course/study requirements |
| Service Development project |

How satisfied are you overall in your current job role?

| *Not at all satisfied* | | | | | | *Extremely satisfied* | | | | | |
| --- | --- | --- | --- | --- | --- | --- | --- | --- | --- | --- | --- |
| 0 | 1 | 2 | 3 | 4 | 5 | | 6 | 7 | 8 | 9 | 10 |
|  |  |  |  |  |  | |  |  |  |  |  |

| Overall how would you rate your satisfaction with the structure of the proram and time for your development and supervision during the AHRG program? | | | | |
| --- | --- | --- | --- | --- |
| *Very dissatisfied* | *Dissatisfied* | *Neither satisfied or dissatisfied* | *Satisfied* | *Very Satisfied* |
|  |  |  |  |  |
| How could the supervision and structure of the program be improved to better support you for the remainder of the AHRGP or for future AHRG trainees? | | | | |
|  | | | | |

How have you found fitting the study into your work life balance

Very difficult, difficult, neutral, easy, very easy

## AHRGP Trainee midway interview questions

What have been the most useful aspects of AHRGP?

What have been the least useful aspects of the AHRGP?

How is supervision going? How about management support? How could these be improved to better support you?

How are you going putting time aside to study? Do you have enough time?

What aspects of the course have you been able to implement into your work? And how/ explain….

Have you noticed any changes to your practice since commencing the AHRGP? If so what changes?

What have been the most challenging aspects of undertaking the JCU modules? What has been the best bits?

Can you briefly outline the service development project that you are participating in as part of your program?

How far along are you in completing this? When do you expect it to be completed?

What have been some of the enablers and barriers to implementing the service development project?

Has this pathway had an impact on your intention to continue to work in a rural and remote location? If so how?

What other factors are impacting on your intention to stay?

## AHRGP Trainee end point survey questions

How satisfied are you in relation to the overall AHRGP?

Not at all satisfied, not satisfied, neutral, satisfied, very satisfied

What were the most useful aspects of the course?

What were the least useful aspects of the course?

| How likely are you to recommend this program to a peer? | | | | |
| --- | --- | --- | --- | --- |
| *Very unlikely* | *Unlikely* | *Neutral* | *Likely* | *Very likely* |
|  |  |  |  |  |

Can you explain why you gave this answer?

What aspects of the course have you been able to implement into your work?

Have you noticed any changes to your practice? If so what changes?

| How satisfied are you overall in your current job role?   \| *Not at all satisfied* \| \| \| \| \| \| *Extremely satisfied* \| \| \| \| \| \| \| --- \| --- \| --- \| --- \| --- \| --- \| --- \| --- \| --- \| --- \| --- \| --- \| \| 0 \| 1 \| 2 \| 3 \| 4 \| 5 \| \| 6 \| 7 \| 8 \| 9 \| 10 \| \|  \|  \|  \|  \|  \|  \| \|  \|  \|  \|  \|  \|   Currently in your role, how would you rate your CONFIDENCE as a rural generalist professional when: | | | | | | | | | | | |
| --- | --- | --- | --- | --- | --- | --- | --- | --- | --- | --- | --- | --- | --- | --- | --- | --- | --- | --- | --- | --- | --- | --- | --- | --- | --- | --- | --- | --- | --- | --- | --- | --- | --- | --- | --- | --- | --- | --- | --- | --- | --- | --- | --- | --- | --- | --- | --- |
| 1. Seeing patients/clients across the age spectrum (e.g. infants, children and adolescents, adults and older people)? | | | | | | | | | | | |
| *Not confident at all* | | | | | | *Extremely confident* | | | | | |
| 0 | 1 | 2 | 3 | 4 | 5 | | 6 | 7 | 8 | 9 | 10 |
|  |  |  |  |  |  | |  |  |  |  |  |
| 1. Delivering a large variety of services (e.g. health promotion, early intervention, acute hospital based services, sub-acute and ambulatory care services, chronic disease management, aged care, palliative care)? | | | | | | | | | | | |
| *Not confident at all* | | | | | | *Extremely confident* | | | | | |
| 0 | 1 | 2 | 3 | 4 | 5 | | 6 | 7 | 8 | 9 | 10 |
|  |  |  |  |  |  | |  |  |  |  |  |
| 1. Working across a variety of health settings (e.g. hospitals, health centres and clinics, patient homes, community venues) | | | | | | | | | | | |
| *Not confident at all* | | | | | | *Extremely confident* | | | | | |
| 0 | 1 | 2 | 3 | 4 | 5 | | 6 | 7 | 8 | 9 | 10 |
|  |  |  |  |  |  | |  |  |  |  |  |
| How would you rate your OVERALL CONFIDENCE as a generalist professional? | | | | | | | | | | | |
| *Not confident at all* | | | | | | *Extremely confident* | | | | | |
| 0 | 1 | 2 | 3 | 4 | 5 | | 6 | 7 | 8 | 9 | 10 |
|  |  |  |  |  |  | |  |  |  |  |  |

Overall how did you find fitting the study into your work life balance

Very difficult, difficult, neutral, easy, very easy

Were you consistently able to allocate a minimum of 0.1-0.2FTE per week? to the program?

| Consider your average working week: approximately how much time per week was dedicated to your: |
| --- |
| Supervision |
| Course/study requirements |
| Service Development project |
| Consider the formal clinical supervision you received as part of this program. Over the duration of your study, did you receive clinical supervision from: |
| Predominantly 1 supervisor for the duration of my study  Predominantly 2 supervisors for the duration of my study  Predominantly 3 supervisors for the duration of my study  More than 3 supervisors for the duration of my study |
| Over the course of your program, what percentage of time was your supervisor: |
| Co-located _________  Remote _________ |

What percentage of your clinical supervision was on-site/face to face vs remote?

How was the remote supervision provided – Video conference, teleconference, face to face visits (what percentage of each)

How would you rate your satisfaction with the clinical supervision you have received?

| *Very dissatisfied* | *Dissatisfied* | *Neither satisfied or dissatisfied* | *Satisfied* | *Very Satisfied* |
| --- | --- | --- | --- | --- |
|  |  |  |  |  |

Did you and your clinical supervisor undertake an evaluation of clinical supervision in the last 12 months? (Yes / No)

What management support did you receive and how satisfied were you with this?

| *Very dissatisfied* | *Dissatisfied* | *Neither satisfied or dissatisfied* | *Satisfied* | *Very Satisfied* |
| --- | --- | --- | --- | --- |
|  |  |  |  |  |

## AHRGP Trainee endpoint interview

Can you tell me what you felt were the most useful components of the AHRGP?

How well do you feel you were able to implement what you learnt in the JCU modules into your practice??

What were the most difficult aspects you had to deal with within your AHRGP program? What helped you over some these?

How was your supervisor and manager support? what impact did they have on your outcomes and experience of the program?

What were some of the unexpected benefits?

What have been some of the enablers and barriers to implementing the service development project?

What were the benefits for you, your team, your organisation, consumers, outreach services, your peers etc

Were you consistently able to allocate a minimum of 0.1-0.2FTE per week to the program?

What impact did the protected minimum 0.1-.2 FTE time allocated to the project have on your usual work role?

How does this impact compare with the benefits of the AHRGP? Was it worth it?

When we met at the beginning of the project you listed the following factors as the most important in impacting on your intention to stay in a rural or remote location. Have these factors or their importance changed since then? If so how

You said at the beginning that you intended to remain in this location for ….. has this changed? If so how and why?

What else could your organisation be doing for allied health professional retention and support?

How could we improve the pathway for future participants?

Did you find this stage of your career to be appropriate for commencing the AHRGP? If not what stage would you consider to be appropriate and why? If yes why was it a good time?

Review personality data

Reflecting on your results what did they show?

Did they reflect your temperament and character?

Did you learn anything new about yourself?

Do these results give us any insight into why you came to a rural area? Why you have stayed? How you have coped? Why you did the study? How you have handled the study?

Do you have any final comments?

## AHRGP Trainee 6 month post survey

Where are you currently working?

What position are you in?

What service are you working for?

What impact did the Allied Heath Rural Generalist Pathway program have on your decision to stay or leave a rural area?

What other factors impacted on your decision to leave or stay?

How long do you intend to stay?

Any other feedback?

## AHRGP Supervisors pre pathway interview

Name

Profession

Local Health Network

Location

Position title

Classification

Did you grow up in a rural or metropolitan centre?

How long have you been working in a rural location?

How long do you intend to work in a rural or remote location?

| 1. Currently in your role as clinical supervisor, how would you rate the trainee’s COMPETENCE as a rural generalist professional when: | | | | | | | | | | | |
| --- | --- | --- | --- | --- | --- | --- | --- | --- | --- | --- | --- |
| 1. Seeing patients/clients across the age spectrum (e.g. infants, children and adolescents, adults and older people)? | | | | | | | | | | | |
| *Not confident at all* | | | | | | *Extremely confident* | | | | | |
| 0 | 1 | 2 | 3 | 4 | 5 | | 6 | 7 | 8 | 9 |  |
|  |  |  |  |  |  | |  |  |  |  |  |
| 1. Delivering a large variety of services (e.g. health promotion, early intervention, acute hospital-based services, sub-acute and ambulatory care services, chronic disease management, aged care, palliative care)? | | | | | | | | | | | |
| *Not confident at all* | | | | | | *Extremely confident* | | | | | |
| 0 | 1 | 2 | 3 | 4 | 5 | | 6 | 7 | 8 | 9 |  |
|  |  |  |  |  |  | |  |  |  |  |  |
| 1. Working across a variety of health settings (e.g. hospitals, health centres and clinics, patient homes, community venues) | | | | | | | | | | | |
| *Not confident at all* | | | | | | *Extremely confident* | | | | | |
| 0 | 1 | 2 | 3 | 4 | 5 | | 6 | 7 | 8 | 9 |  |
|  |  |  |  |  |  | |  |  |  |  |  |
| 1. How would you rate the trainees OVERALL CONFIDENCE as a generalist professional? | | | | | | | | | | | |
| *Not confident at all* | | | | | | *Extremely confident* | | | | | |
| 0 | 1 | 2 | 3 | 4 | 5 | | 6 | 7 | 8 | 9 |  |
|  |  |  |  |  |  | |  |  |  |  |  |

What profession(s) are you supporting in the allied health rural generalist pathway program?

Occupational Therapy, Physiotherapy, Podiatry, Speech pathology

How many trainees are you providing clinical supervision to?

What has been your previous experience of working with early career AHPs? (has it been positive/negative if so why/why not etc)

In your opinion, what are some common challenges and opportunities for early career allied health professionals working in rural and remote areas?

How do these challenges and opportunities impact on recruitment and retention of AHPs?

How do you anticipate the trainee’s involvement in this program will support or improve allied health practice within your profession and/or health service?

What do you see consumers need from allied health services that the trainees will be able to better support following completion of the program?

What impact do you anticipate this pathway will have for

1. the AHPs involved
2. the allied health workforce
3. the organisation

What do you think the enablers and barriers will be in achieving outcomes from the AHRGP?

What are some of the supports you anticipate you will provide to trainees during this program?

How do you anticipate your workload will be impacted by the program? Do you think there will be any other impacts on your role?

What strategies are in place to ensure minimum 0.1- 0.2FTE of workload is allocated for the trainee’s participation in the program?

What do you see as the personal, organisational and professional factors that impact on retention in your location and/or profession?

How would you rate or describe these factors in terms of most impacting versus least impacting?

## AHRGP Supervisors midway interview

Before we get started, to give some context to the answers you provide;

Can you comment on how far along in the program the trainee you are supporting is? i.e. how many modules have they done, how many are they doing at the moment etc.

Have they been involved in a service delivery project yet and if so what has that been?

Is the trainee you are working with in level 1 or level 2 of the program?

Can you tell me a bit about why the trainee was selected? What factors did you consider in selecting trainees?

What changes in practice have you noticed in the AHP trainees? (changes to work capacity, performance, confidence, satisfaction etc)

What changes are others noticing in the AHP trainees? Other staff, consumers? (capacity, quality/ performance, confidence, autonomy)

Has the program been discussed with consumers? Do consumers know if their treating clinician is participating in the program? If so how?

How do you think the trainee’s participation in the program has impacted consumers? i.e reduced travel time, better access to services, reduced wait times/earlier access to services, increased multi d support, better communication between team members, increased service capacity/ reduced complexity of services/less professionals involved, improved care?

What aspects of the pathway program have worked well in your setting and profession?

What has not been working well?

What have been the main challenges in supervising a trainee?

What advantages are there in supervising a trainee?

How much time on average per week have you spent in clinical supervision with the trainee?

What has been unexpected in the process?

What further changes to the trainee’s practice are you anticipating? Or hoping for?

At what stage in an AHPs career do you think is a good time for trainees to undertake the program?

In these early stages, what are your thoughts about whether this program is suiting the needs of your profession?

Do you think this program suits this location where the trainee is based? Why/why not

What sort of trainee personal attributes do you think this program suits?

Would you supervise a trainee again?

| How likely are you to recommend this program to others? | | | | |
| --- | --- | --- | --- | --- |
| *Very unlikely* | *Unlikely* | *Neutral* | *Likely* | *Very likely* |
|  |  |  |  |  |

Answer this relevant to the trainee involved: (post)

| Currently in your role, how would you rate your trainee’s COMPETENCE as a rural generalist professional when: | | | | | | | | | | | |
| --- | --- | --- | --- | --- | --- | --- | --- | --- | --- | --- | --- |
| 1. Seeing patients/clients across the age spectrum (e.g. infants, children and adolescents, adults and older people)? | | | | | | | | | | | |
| *Not confident at all* | | | | | | *Extremely confident* | | | | | |
| 0 | 1 | 2 | 3 | 4 | 5 | | 6 | 7 | 8 | 9 |  |
|  |  |  |  |  |  | |  |  |  |  |  |
| Delivering a large variety of services (e.g. health promotion, early intervention, acute hospital based services, sub-acute and ambulatory care services, chronic disease management, aged care, palliative care)? | | | | | | | | | | | |
| *Not confident at all* | | | | | | *Extremely confident* | | | | | |
| 0 | 1 | 2 | 3 | 4 | 5 | | 6 | 7 | 8 | 9 |  |
|  |  |  |  |  |  | |  |  |  |  |  |
| Working across a variety of health settings (e.g. hospitals, health centres and clinics, patient homes, community venues) | | | | | | | | | | | |
| *Not confident at all* | | | | | | *Extremely confident* | | | | | |
| 0 | 1 | 2 | 3 | 4 | 5 | | 6 | 7 | 8 | 9 |  |
|  |  |  |  |  |  | |  |  |  |  |  |
| How would you rate your trainee’s OVERALL CONFIDENCE as a generalist professional? | | | | | | | | | | | |
| *Not confident at all* | | | | | | *Extremely confident* | | | | | |
| 0 | 1 | 2 | 3 | 4 | 5 | | 6 | 7 | 8 | 9 |  |
|  |  |  |  |  |  | |  |  |  |  |  |

## AHRGP Supervisors post interview

Before we get started, to give some context to the answers you provide;

Please comment on whether the trainee you were working with completed the program? If not how many modules were completed?

Was a service delivery project completed and if so what was the focus?

The last time we spoke, you were anticipating the following changes to practice……., did these eventuate?

What other changes to practice did you notice? (changes to work capacity, performance/ competence, confidence, satisfaction etc)

What other changes did others anecdotally report to you? (capacity, quality/ performance, confidence, autonomy)

Review of answers from midway interview and check to see if further changes have been noticed, if perceptions have changed:

How do you think the trainee’s participation in the program impacted consumers? i.e reduced travel time, better access to services, reduced wait times/earlier access to services, increased multi d support, better communication between team members, increased service capacity/ reduced complexity of services/less professionals involved?

What aspects of the pathway program worked well in your setting and profession?

What did not work well?

What were the main challenges in supervising a trainee?

What were the advantages in supervising a trainee?

How much time on average per week did you spend in clinical supervision with the trainee?

What was unexpected in the process?

Answer this relevant to the trainee involved:

| Currently in your role, how would you rate your trainee’s COMPETENCE as a rural generalist professional when: | | | | | | | | | | | |
| --- | --- | --- | --- | --- | --- | --- | --- | --- | --- | --- | --- |
| 1. Seeing patients/clients across the age spectrum (e.g. infants, children and adolescents, adults and older people)? | | | | | | | | | | | |
| *Not confident at all* | | | | | | *Extremely confident* | | | | | |
| 0 | 1 | 2 | 3 | 4 | 5 | | 6 | 7 | 8 | 9 |  |
|  |  |  |  |  |  | |  |  |  |  |  |
| Delivering a large variety of services (e.g. health promotion, early intervention, acute hospital based services, sub-acute and ambulatory care services, chronic disease management, aged care, palliative care)? | | | | | | | | | | | |
| *Not confident at all* | | | | | | *Extremely confident* | | | | | |
| 0 | 1 | 2 | 3 | 4 | 5 | | 6 | 7 | 8 | 9 |  |
|  |  |  |  |  |  | |  |  |  |  |  |
| Working across a variety of health settings (e.g. hospitals, health centres and clinics, patient homes, community venues) | | | | | | | | | | | |
| *Not confident at all* | | | | | | *Extremely confident* | | | | | |
| 0 | 1 | 2 | 3 | 4 | 5 | | 6 | 7 | 8 | 9 |  |
|  |  |  |  |  |  | |  |  |  |  |  |
| How would you rate their OVERALL CONFIDENCE as a generalist professional? | | | | | | | | | | | |
| *Not confident at all* | | | | | | *Extremely confident* | | | | | |
| 0 | 1 | 2 | 3 | 4 | 5 | | 6 | 7 | 8 | 9 |  |
|  |  |  |  |  |  | |  |  |  |  |  |

At what stage in an AHPs career do you think is a good time for trainees to undertake the program?

What are your thoughts about whether this program is suiting the needs of your profession?

Do you think this program suits this location where the trainee is based? Why/why not

What sort of trainee personal attributes do you think this program suits?

Do you have any recommendations about the process for selection or recruitment of trainees?

Would you supervise a trainee again?

| How likely are you to recommend this program to others? | | | | |
| --- | --- | --- | --- | --- |
| *Very unlikely* | *Unlikely* | *Neutral* | *Likely* | *Very likely* |
|  |  |  |  |  |

## AHRGP Clinical lead pre pathway interview

Name

Profession

Local Health Network

Location

Position title

Classification

Did you grow up in a rural or metropolitan centre?

How long have you been working in a rural or remote location?

How long do you intend to work in a rural or remote location?

How many AHRGP trainees are you supporting in your profession?

What impact do you anticipate this pathway will have for the:

1. AHPs involved
2. allied health workforce
3. organisation

How do you think your role will be impacted by this program?

What do you think the challenges will be?

What outcomes are you most hoping will be achieved through trainee’s participation in the program?

What do you think the enablers and barriers will be in achieving outcomes?

What do you see consumers need from allied health services that the trainees will be able to better support following completion of the program?

What has been your experience of working with early career AHPs?

How long do AHPs from your profession stay in CHSA on average?

What is it like recruiting to AHP positions in your profession?

What is your role in clinical supervision of AHPs?

What is your role in service development activities or continuous quality improvement?

What are the roles of allied health professions in your profession in rural and remote areas? How are they different to your metropolitan counterparts?

In your opinion, what are the challenges and opportunities of early career allied health professionals working in rural and remote areas?

How do these challenges and opportunities impact on recruitment and retention of AHPs?

What do you see as the personal, organisational and professional factors that impact on retention in rural areas in your profession?

How would you rate or describe these in terms of most impacting versus least impacting?

## AHRGP Clinical lead midway interview

Can you tell me a bit about why the trainee was selected? What factors did you consider in selecting trainees?

What changes in practice have you noticed in the AHP trainees?

What changes in the AHP trainees are others noticing? Other staff, consumers?

How do you think the program has impacted consumers? - i.e reduced travel time, better access to services, reduced wait times/earlier access to services, increased multi d support, better communication between team members, increased service capacity/ reduced complexity of services/less professionals involved?

What has worked well in your profession?

What has not worked well?

What have been the main challenges?

Were you aware of strategies being in place to support a minimum of 0.1- 0.2FTE of the trainees workload being allocated to the program? If so have these been effective?

Have there been any difficulties providing ACL support to the trainees from your profession?

What has been unexpected in the process?

What further changes to practice in the trainees are you anticipating? Or hoping for?

Do you think the AHRGP is suitable for your profession?

Which rural locations do you think this program suits?

What sort of trainee personal attributes do you think this program suits?

When during an AHPs career do you think is a good time for trainees to undertake the pathway?

Would you support a trainee again?

| 1. How likely are you to recommend this program to others? | | | | |
| --- | --- | --- | --- | --- |
| *Very unlikely* | *Unlikely* | *Neutral* | *Likely* | *Very likely* |
|  |  |  |  |  |

## AHRGP Clinical lead post interview

The last time we spoke, you were anticipating the following changes to practice……., did these eventuate?

What other changes to practice did you notice? (changes to work capacity, confidence, satisfaction, performance etc)

What other changes did others anecdotally report to you? (capacity, quality, confidence, autonomy, performance)

Review of answers from midway interview and check to see if further changes have been noticed, if perceptions have changed:

How do you think trainees participation in the program impacted consumers? i.e reduced travel time, better access to services, reduced wait times/earlier access to services, increased multi d support, better communication between team members, increased service capacity/ reduced complexity of services/less professionals involved?

What aspects of the pathway program worked well in your profession?

What did not work well?

What were the main challenges you faced in your role with the program?

What were the advantages for your role with the program?

What was unexpected in the process?

At what stage in an AHPs career do you think is a good time for trainees to undertake the program?

What are your thoughts about whether your profession suits this program?

Do you think this program suits this location where the trainee were based? Why/why not

What sort of trainee personal attributes do you think this program suits?

Do you have any recommendations about the process for selection or recruitment of trainees?

Would you want to be involved in the program again?

| How likely are you to recommend this program to others? | | | | |
| --- | --- | --- | --- | --- |
| *Very unlikely* | *Unlikely* | *Neutral* | *Likely* | *Very likely* |
|  |  |  |  |  |

## AHRGP Manager pre pathway interview

Name

Profession

Local Health Network

Location

Position title

Classification

Did you grow up in a rural or metropolitan centre?

How long have you been working in a rural or remote location?

How long do you intend to work in a rural or remote location?

| Currently in your role, how would you rate the trainee’s COMPETENCE as a rural generalist professional when: | | | | | | | | | | | |
| --- | --- | --- | --- | --- | --- | --- | --- | --- | --- | --- | --- |
| 1. Seeing patients/clients across the age spectrum (e.g. infants, children and adolescents, adults and older people)? | | | | | | | | | | | |
| *Not confident at all* | | | | | | *Extremely confident* | | | | | |
| 0 | 1 | 2 | 3 | 4 | 5 | | 6 | 7 | 8 | 9 |  |
|  |  |  |  |  |  | |  |  |  |  |  |
| 1. Delivering a large variety of services (e.g. health promotion, early intervention, acute hospital based services, sub-acute and ambulatory care services, chronic disease management, aged care, palliative care)? | | | | | | | | | | | |
| *Not confident at all* | | | | | | *Extremely confident* | | | | | |
| 0 | 1 | 2 | 3 | 4 | 5 | | 6 | 7 | 8 | 9 |  |
|  |  |  |  |  |  | |  |  |  |  |  |
| 1. Working across a variety of health settings (e.g. hospitals, health centres and clinics, patient homes, community venues) | | | | | | | | | | | |
| *Not confident at all* | | | | | | *Extremely confident* | | | | | |
| 0 | 1 | 2 | 3 | 4 | 5 | | 6 | 7 | 8 | 9 |  |
|  |  |  |  |  |  | |  |  |  |  |  |
| How would you rate the trainees OVERALL CONFIDENCE as a generalist professional? | | | | | | | | | | | |
| *Not confident at all* | | | | | | *Extremely confident* | | | | | |
| 0 | 1 | 2 | 3 | 4 | 5 | | 6 | 7 | 8 | 9 |  |
|  |  |  |  |  |  | |  |  |  |  |  |

What profession(s) are you supporting in the allied health rural generalist pathway program?

Occupational Therapy, Physiotherapy, Podiatry, Speech pathology

How many trainees are you line managing to?

What has been your experience of working with early career AHPs?

In your opinion, what are the challenges and opportunities experienced by early career allied health professionals working in rural and remote areas?

How do these challenges and opportunities impact on recruitment and retention of AHPs?

How do you anticipate the trainee’s involvement in this program will support or improve allied health practice?

What do you see consumers need from allied health services that the trainees will be able to better support following completion of the program?

What do you see as the personal, organisational and professional factors that impact on retention in your location and/or profession?

How would you rate or describe these factors in terms of most impacting versus least impacting?

What impact do you anticipate this pathway will have for the

1. AHPs involved
2. allied health workforce
3. The organisation

What do you think the enablers and barriers will be in achieving these?

What are some of the roles you anticipate you will have during this program?

How do you anticipate your workload will be impacted by your LHN’s trainees’ participation in the program?

What strategies are in place to ensure minimum 0.1- 0.2FTE of workload is allocated for the trainee’s participation in the program?

## AHRGP Manager midway interview

Can you tell me a bit about why the trainee was selected? What factors did you consider in selecting trainees?

What changes in practice have you noticed in the AHP trainees? (changes to work capacity, confidence, satisfaction, performance etc)

What changes are others noticing in the AHP trainees? Other staff, consumers? (capacity, quality, confidence, autonomy, performance)

Has the program been discussed with consumers? Do consumers know if their treating clinician is participating in the program? If so how?

How do you think the trainee’s participation in the program has impacted consumers? i.e reduced travel time, better access to services, reduced wait times/earlier access to services, increased multi d support, better communication between team members, increased service capacity/ reduced complexity of services/less professionals involved?

What has worked well in your LHN?

What has not worked well?

What have been the main challenges?

Was the trainee consistently able to allocate a minimum of 0.1-0.2FTE to the program?

What was the impact of loss of 0.1-0.2FTE allocated to the program? How did this rate against the benefits seen from the trainee’s participation in the project?

How have you found line managing a trainee? What has it been like? What has been unexpected in the process?

On average how much time each week do you spend supporting trainees or contributing to the AHRGP process?

What further changes to the trainees’ practice are you anticipating? Or hoping for?

In these early stages, what are your thoughts about what professions you think this program suits?

Do you think this program suits your locations? Why/why not?

At what stage in an AHPs career do you think is a good time for trainees to undertake the program?

What sort of trainee personal attributes do you think this program suits?

Would you line manage a trainee again?

| How likely are you to recommend this program to others? | | | | |
| --- | --- | --- | --- | --- |
| *Very unlikely* | *Unlikely* | *Neutral* | *Likely* | *Very likely* |
|  |  |  |  |  |

Answer this relevant to the trainee involved: (post)

| Currently in your role, how would you rate the COMPETENCE of your trainee as a rural generalist professional when: | | | | | | | | | | | |
| --- | --- | --- | --- | --- | --- | --- | --- | --- | --- | --- | --- |
| 1. Seeing patients/clients across the age spectrum (e.g. infants, children and adolescents, adults and older people)? | | | | | | | | | | | |
| *Not confident at all* | | | | | | *Extremely confident* | | | | | |
| 0 | 1 | 2 | 3 | 4 | 5 | | 6 | 7 | 8 | 9 |  |
|  |  |  |  |  |  | |  |  |  |  |  |
| 1. Delivering a large variety of services (e.g. health promotion, early intervention, acute hospital based services, sub-acute and ambulatory care services, chronic disease management, aged care, palliative care)? | | | | | | | | | | | |
| *Not confident at all* | | | | | | *Extremely confident* | | | | | |
| 0 | 1 | 2 | 3 | 4 | 5 | | 6 | 7 | 8 | 9 |  |
|  |  |  |  |  |  | |  |  |  |  |  |
| 1. Working across a variety of health settings (e.g. hospitals, health centres and clinics, patient homes, community venues) | | | | | | | | | | | |
| *Not confident at all* | | | | | | *Extremely confident* | | | | | |
| 0 | 1 | 2 | 3 | 4 | 5 | | 6 | 7 | 8 | 9 |  |
|  |  |  |  |  |  | |  |  |  |  |  |
| How would you rate their OVERALL CONFIDENCE as a generalist professional? | | | | | | | | | | | |
| *Not confident at all* | | | | | | *Extremely confident* | | | | | |
| 0 | 1 | 2 | 3 | 4 | 5 | | 6 | 7 | 8 | 9 |  |
|  |  |  |  |  |  | |  |  |  |  |  |

## AHRGP Manager post interview

Before we get started, to give some context to the answers you provide;

Please comment on whether the trainee you were working with completed the program? If not how many modules were completed?

Was a service delivery project completed and if so what was the focus?

Was the trainee you worked with in level 1 or level 2 of the program?

The last time we spoke, you were anticipating the following changes to practice……., did these eventuate?

What other changes to practice did you notice? (changes to work capacity, confidence, satisfaction, performance etc)

What other changes did others anecdotally report to you? (capacity, quality, confidence, autonomy, performance )

How do you think the trainee’s participation in the program impacted consumers? i.e reduced travel time, better access to services, reduced wait times/earlier access to services, increased multi d support, better communication between team members, increased service capacity/ reduced complexity of services/less professionals involved?

What aspects of the pathway program worked well in your setting?

What did not work well?

What were the main challenges in line-managing a trainee?

What were the advantages in line-managing a trainee?

How much time on average per week did you spend supporting the trainee?

What was unexpected in the process?

Answer this relevant to the trainee involved: (post)

| Currently in your role, how would you rate your trainee’s COMPETENCE as a rural generalist professional when: | | | | | | | | | | | |
| --- | --- | --- | --- | --- | --- | --- | --- | --- | --- | --- | --- |
| 1. Seeing patients/clients across the age spectrum (e.g. infants, children and adolescents, adults and older people)? | | | | | | | | | | | |
| *Not confident at all* | | | | | | *Extremely confident* | | | | | |
| 0 | 1 | 2 | 3 | 4 | 5 | | 6 | 7 | 8 | 9 |  |
|  |  |  |  |  |  | |  |  |  |  |  |
| Delivering a large variety of services (e.g. health promotion, early intervention, acute hospital based services, sub-acute and ambulatory care services, chronic disease management, aged care, palliative care)? | | | | | | | | | | | |
| *Not confident at all* | | | | | | *Extremely confident* | | | | | |
| 0 | 1 | 2 | 3 | 4 | 5 | | 6 | 7 | 8 | 9 |  |
|  |  |  |  |  |  | |  |  |  |  |  |
| Working across a variety of health settings (e.g. hospitals, health centres and clinics, patient homes, community venues) | | | | | | | | | | | |
| *Not confident at all* | | | | | | *Extremely confident* | | | | | |
| 0 | 1 | 2 | 3 | 4 | 5 | | 6 | 7 | 8 | 9 |  |
|  |  |  |  |  |  | |  |  |  |  |  |
| How would you rate their OVERALL CONFIDENCE as a generalist professional? | | | | | | | | | | | |
| *Not confident at all* | | | | | | *Extremely confident* | | | | | |
| 0 | 1 | 2 | 3 | 4 | 5 | | 6 | 7 | 8 | 9 |  |
|  |  |  |  |  |  | |  |  |  |  |  |

At what stage in an AHPs career do you think is a good time for trainees to undertake the program?

What are your thoughts about what professions this program suits?

Do you think this program suits this location where the trainee is based? Why/why not

What sort of trainee personal attributes do you think this program suits?

Do you have any recommendations about the process for selection or recruitment of trainees?

Would you want to manage a trainee again?

| How likely are you to recommend this program to others? | | | | |
| --- | --- | --- | --- | --- |
| *Very unlikely* | *Unlikely* | *Neutral* | *Likely* | *Very likely* |
|  |  |  |  |  |

## AHRGP Project managers pre interview

Can you please provide us with the following information to assist us in understanding the costs of the program and projected benefits:

Number of participants – level 1/level 2

Cost of tuition – level 1/level 2

Projected time trainees are allocated in their workload to complete training and associated activities

Are there any travel costs associated with the training?

Can you provide us with some information about retention rates in rural and remote locations in SA? How long do early career AHP’s stay in general?

We will be asking the supervisors, managers and trainees about how much time they are spending on the program as well, can you think of any other costs that will be associated with the AHRGP? Include costs of centralised coordination

What are the intentions of the rural generalist pathway?

What are the aims, objectives and potential outcomes of the program?

What will the community see as different?

What will consumers experience as a benefit?

What is the timeframe for implementation?

When will the trainees start and finish?

How many allied health professionals (AHPs) currently work in Country Health SA (CHSA)?

How many trainees (& which professions) have been recruited into each of the two programs?

What is the current retention rate for AHPs in CHSA?

What satisfaction data already exists?

How are community/consumer needs determined?

How are workforce configurations planned around community needs?

How do we know what professions are required for a health service, how is this reviewed?

What is the role of workforce planning and how to they consider community need and allied health?

How is quality measured in CHSA? What is measured?

What is a quality service or outcome and how will this be measured in the project?

## AHRGP Program managers midway/end point interview

Review projected costs from survey and discuss any changes that may have occurred

What goals have the program achieved (that were set out at the beginning)?

What worked well? What didn’t? (program as well as service challenges and benefits)

Who was in the program, what were their demographics etc?

What disciplines? Does this fit with the demographics in SA?

What has been the retention rate of trainees within the program?
vs the average AHP retention rate?

What were the demographics of any trainees that withdrew from the program? (contract status, nomination status, reasons provided for withdrawal)

What have been the short-term impacts of the program? For trainees, for supervisors/managers, for host sites, for consumers, for communities, what has been outcomes have been achieved?

Can you tell me more about the service development projects that the trainees were involved in? from a central viewpoint how did they go overall? What were some of the benefits that were achieved? Were there any costs?

What is the future of the service development projects? Will they continue? Were they worth pursuing? What were some of the contributing factors to their success or failure?

What did the program cost overall? Tuition fees, FTE, other costs

Was the budget met?

Would you recommend continuing the program? Why/why not?

What are the demographics of the sites that it worked well in?

What professions worked well in SA?

What sorts of clinicians/personal attributes work well as trainees?

What is the plan for sustainability of the program? How could it be funded in the future?

## AHRGP Consumer representatives pre program focus group

As representative of consumers, what would you consider the features of high quality allied health services are?

What would consumers like allied health clinicians to be focusing on?

How can allied health professionals assist in meeting the needs of rural and remote areas?

We have occupational therapy, physiotherapy, podiatry, speech pathology trainees participating in this program.

From your understanding as a consumer representative of your local area, what would consumers consider as some of the positive attributes of these professions?

What would consumers consider some of the negative attributes of these professions for your local areas?

As a representative of consumers, what specifically would you like these professions to be aware of when delivering quality services? Break down into separate professions

## AHRGP Consumer representative post program focus group

Since we met last time the allied health trainees have completed or partially completed their studies and the pathway.

When we first met you talked about the following positive and negative attributes of allied health professions from consumers perspectives in rural South Australia.

Are these attributes still relevant for consumes in your communities? If relevant what other attributes of allied health professions would consumers in rural South Australia describe?

These are the sorts of changes we are seeing happening as a result of the program (list service development projects)

Considering the changes that this program has seen in rural South Australia, which changes would you consider are important for consumers that you represent? Why would consumers see these as important?
